# Thermodynamic Properties of the First-Generation Hybrid Dendrimer with “Carbosilane Core/Phenylene Shell” Structure

**DOI:** 10.3390/e23121557

**Published:** 2021-11-23

**Authors:** Semen S. Sologubov, Alexey V. Markin, Natalia N. Smirnova, Elena S. Chamkina, Irina Yu. Krasnova, Sergey A. Milenin, Olga A. Serenko, Zinaida B. Shifrina, Aziz M. Muzafarov

**Affiliations:** 1 Chemistry Department, National Research Lobachevsky State University of Nizhny Novgorod, 23/5 Gagarin Av., 603950 Nizhny Novgorod, Russia; s.slg90@gmail.com (S.S.S.); smirnova@ichem.unn.ru (N.N.S.); 2 Nesmeyanov Institute of Organoelement Compounds of Russian Academy of Sciences, 28 Vavilov St., 119334 Moscow, Russia; elena.serkova@ineos.ac.ru (E.S.C.); irina7krasnova@yandex.ru (I.Y.K.); oserenko@yandex.ru (O.A.S.); z_shifrina@yahoo.com (Z.B.S.); aziz@ispm.ru (A.M.M.); 3 Enikolopov Institute of Synthetic Polymeric Materials of Russian Academy of Sciences, 70 Profsoyuznaya St., 117393 Moscow, Russia; cephe@mail.ru

**Keywords:** hybrid dendrimers, adiabatic calorimetry, DSC, heat capacity, glass transition, thermodynamic functions

## Abstract

The molar heat capacity of the first-generation hybrid dendrimer with a “carbosilane core/phenylene shell” structure was measured for the first time in the temperature range *T* = 6–600 K using a precise adiabatic vacuum calorimeter and DSC. In the above temperature interval, the glass transition of the studied compound was observed, and its thermodynamic characteristics were determined. The standard thermodynamic functions (the enthalpy, the entropy, and the Gibbs energy) of the hybrid dendrimer were calculated over the range from *T* = 0 to 600 K using the experimentally determined heat capacity. The standard entropy of formation of the investigated dendrimer was evaluated at *T* = 298.15 K. The obtained thermodynamic properties of the studied hybrid dendrimer were compared and discussed with the literature data for some of the first-generation organosilicon and pyridylphenylene dendrimers.

## 1. Introduction

Dendrimers are a unique class of perfect monodisperse macromolecules having a highly branched three-dimensional architecture. The well-defined chemical structure of dendrimers consists of three major components: a central core (the multifunctional atomic group), an inner sphere (tree-like branches organized in generations G1, G2, G3, etc.), and an outer shell (comprising a large number of terminal functional groups) [1,2,3]. These structural features of dendrimers lead to their versatile physical and chemical properties, as well as contribute to their applications in nanotechnologies, biomedical fields (anticancer vaccines, contrast agents in magnetic resonance imaging, nanocarriers in targeted drug delivery, and gene therapy), electronics, and catalysis [4,5,6,7,8,9,10,11,12]. In contrast to traditional polymers, dendrimers are synthesized in a stepwise controlled manner. They can be constructed by two distinct synthetic strategies, namely, divergent and convergent approaches. The structural evolution of dendrimers is inextricably associated with a continuous improvement in traditional synthetic strategies, as well as the development of new synthetic tools for fundamental discoveries and practical applications [13].

Among various dendritic macromolecules, carbosilane (Si–C) dendrimers represent one of the most important and promising classes of silicon-containing dendrimers [14,15,16]. These compounds exhibit high flexibility, manifested by low glass transition temperatures, biological inertness, and kinetic and thermodynamic stability due to the low polarity and high strength of the Si–C bond. Therefore, carbosilane dendrimers have attracted much attention as functional molecules for successful applications in electrochemistry, catalysis, and nanoengineering [17]. Polyphenylene dendrimers are highly branched monodisperse macromolecules entirely consisting of substituted benzene rings that result in their rigid, shape-persistent chemical structure [18,19,20,21]. These dendrimers can be synthetically modified by the introduction of different chemical functionalities that influence their applications [22,23]. Polyphenylene dendrimers are extremely chemical and thermal stable molecules, but with greatly increased solubility in a wide range of organic solvents [24,25]. These advantages lead to their perspective applications as active materials in OLED devices, templates for composite materials, molecular sensors, magnetically recoverable catalysts, and bioapplications [26,27,28,29,30,31,32,33,34].

A new direction in dendrimer chemistry can be attributed to the synthesis and study of physicochemical properties of hybrid dendrimers consisting of two chemically different dendritic segments, each of which has an independent highly branched molecular architecture. The arrangement of the dendritic blocks in a different manner (i.e., a flexible core and a rigid shell or vice versa) allows for a controllable synthesis of principally new dendrimers with the desired characteristics [35]. The results of our calorimetric investigations of the widespread families such as carbosilane [36,37,38], siloxane [39,40,41], and polyphenylene [42,43] dendrimers with different molecular structure of the inner sphere and the surface functional groups were published earlier.

The thermodynamic properties of the first-generation hybrid dendrimer with a “carbosilane core/phenylene shell” structure have not yet been studied. These are the key data for technological and thermophysical calculations of processes with this dendrimer. The accumulation of reliable thermodynamic values for this class of macromolecular nanoobjects will subsequently make it possible to obtain practically important “structure–property” dependences. It allows predicting the physicochemical properties of dendrimer molecules that have not yet been studied.

As a continuation of publications on thermodynamics of dendrimers, the present study is dedicated to the calorimetric determination of the molar heat capacity of the first-generation hybrid dendrimer with a “carbosilane core/phenylene shell” structure, as well as the calculation of the standard thermodynamic functions (Δ*H*°, Δ*S*°, and Δ*G*°) in the range of *T* = 6–600 K and the estimation of the standard entropy of its formation at *T* = 298.15 K from the obtained experimental data. A comparative analysis of thermodynamic properties of the investigated hybrid dendrimer with the literature data for the previously studied organosilicon (carbosilane [36,37,38] and siloxane [39]) and pyridylphenylene dendrimers [42,43] of the first generation was performed to develop the fundamental principles of the regulation of different properties of dendrimers by varying their molecular parameters.

## 2. Experimental

### 2.1. Sample

**Figure 1:**
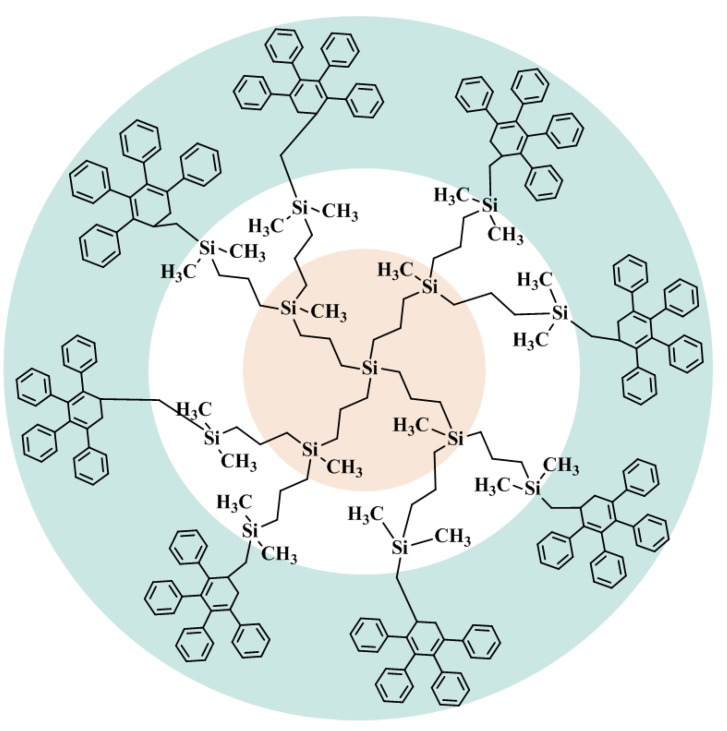
Molecular structure of the first-generation hybrid dendrimer with a “carbosilane core/phenylene shell” structure, G1{Si}_13_[Ar]_32_.

The first-generation hybrid dendrimer with a “carbosilane core/phenylene shell” structure is denoted as G1{Si}_13_[Ar]_32_**,** where G1 indicates the generation number of dendrimer, {Si}_13_ represents the number of silicon atoms in the inner sphere of dendrimer, and [Ar]_32_ corresponds to the number of phenylene units on the outer layer of dendrimer. The molecular structure of the studied dendrimer is presented in Figure 1.

The sample of the hybrid dendrimer G1{Si}_13_[Ar]_32_ was synthesized at the Nesmeyanov Institute of Organoelement Compounds of Russian Academy of Sciences (Moscow) via the Diels–Alder cycloaddition reaction according to the procedure described in detail elsewhere [44]. The synthesized compound was a light-brown powder under the standard conditions and characterized by monodispersity on the generation number. The structure and the composition of dendrimer G1{Si}_13_[Ar]_32_ were confirmed by elemental analysis, ^1^H-, ^13^C-, and ^29^Si-NMR spectroscopy (Bruker AVANCE III HD spectrometer; 500.13 MHz for ^1^H; 125.76 MHz for ^13^C; CDCl_3_ was used as a solvent), and MALDI-TOF mass spectrometry (Bruker BIFLEX III spectrometer; a 337 nm nitrogen laser; indole-3-acrylic acid was used as a matrix, and silver trifluoroacetate was used as a cationization agent). The elemental analysis results for G1{Si}_13_[Ar]_32_ (brutto formula C_304_H_332_Si_13_), reported in [44], are as follows:

- Found (%): C, 83.18; H, 7.51; Si, 8.41.
- Calculated (%): C, 83.92; H, 7.69; Si, 8.39.

The sample of dendrimer G1{Si}_13_[Ar]_32_ was purified by gel permeation chromatography (GPC) in tetrahydrofuran (THF) using a Shimadzu system. According to the GPC analysis results, reported in [44], the final purity of dendrimer G1{Si}_13_[Ar]_32_ was 98%, and the yield of the pure compound was 0.45 g (82%).

The molar mass of dendrimer G1{Si}_13_[Ar]_32_ (*M* = 4350.95 g·mol^−1^) was calculated using the IUPAC table of atomic masses [45]. The sample information for dendrimer G1{Si}_13_[Ar]_32_ is summarized in Table 1.

**Figure 2:**
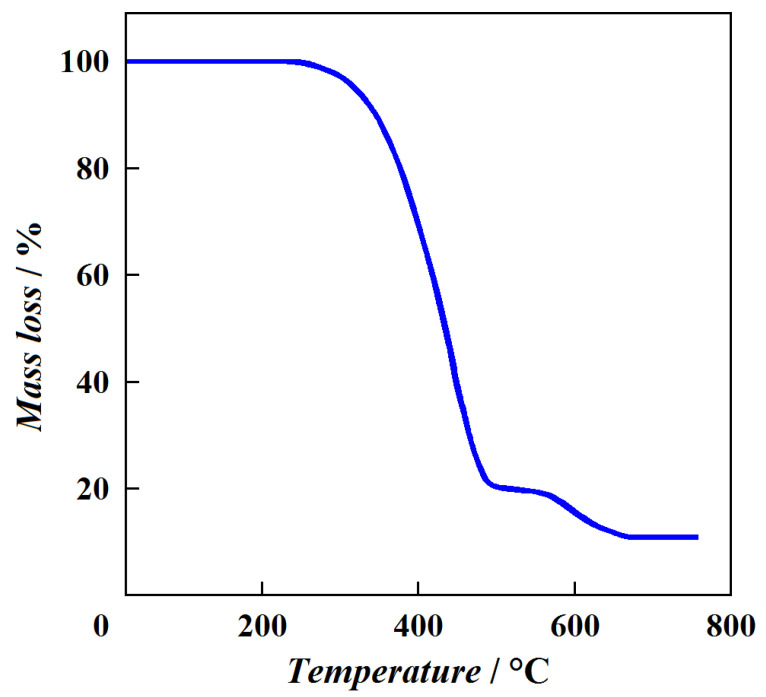
Thermogravimetric curve of hybrid dendrimer G1{Si}_13_[Ar]_32_ in air.

**Figure 3:**
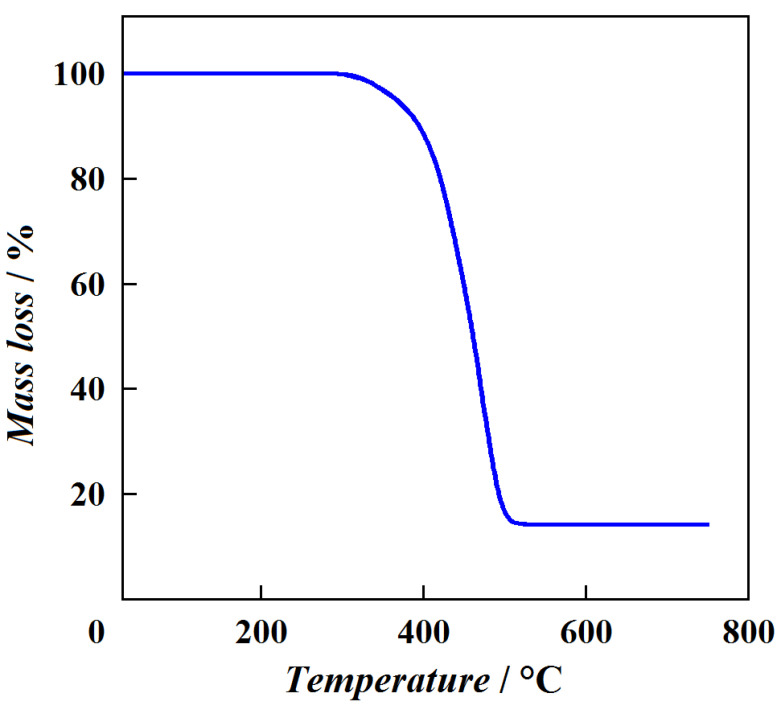
Thermogravimetric curve of hybrid dendrimer G1{Si}_13_[Ar]_32_ in argon.

The preliminary DSC measurements confirmed that the presence of phenylene fragments on the outer layer of hybrid dendrimer G1{Si}_13_[Ar]_32_ significantly increases the glass transition temperature compared to the entirely carbosilane dendrimers (Table 2). The thermal stability of hybrid dendrimer G1{Si}_13_[Ar]_32_ was studied using TG analysis. The decomposition process of the investigated sample in air is illustrated in Figure 2 and described by two steps: the destruction of the carbosilane skeleton (570–670 K) and the decomposition of the aromatic shell (770–970 K). On the contrary, a one-step mass loss (620–820 K), observed during the thermal decomposition of dendrimer G1{Si}_13_[Ar]_32_ in argon, is shown in Figure 3. As a result, the studied sample of hybrid dendrimer G1{Si}_13_[Ar]_32_ is thermally stable up to *T* ~620 K. The difference in TG curves is caused by the oxidation contribution in the decomposition process of the studied hybrid dendrimer. The results of DSC and TG analyses show that the surface layer of hybrid dendrimers strongly influences their thermal properties. In particular, the rigid phenylene shell considerably increases the glass transition temperature of hybrid dendrimers and makes them more thermally stable macromolecules than carbosilane and siloxane dendrimers.

### 2.2. Apparatus and Measurement Procedure

#### 2.2.1. Adiabatic Vacuum Calorimetry

The heat capacity of the hybrid dendrimer with a “carbosilane core/phenylene shell” structure G1{Si}_13_[Ar]_32_ was measured using a thoroughly automated precise adiabatic calorimeter in the temperature range *T* = 6–350 K. The calorimeter construction and the operation procedure were described in detail elsewhere [46]. The calorimetric titanium cell (*V* = 1 cm^3^), containing the studied sample of dendrimer (*m* = 0.1989 g), was kept in vacuum and then filled with gaseous helium for improving thermal conduction. For the temperature measurements, a miniature iron–rhodium resistance thermometer (*R* = 100 Ω), calibrated according to the International Temperature Scale ITS-90, was used; the standard uncertainty for the temperature *u*(*T*) = 0.01 K. The temperature difference between the calorimeter and the adiabatic shield was measured using a four-junction copper-iron/chromel thermocouple. Liquid nitrogen and liquid helium were used in the low-temperature (*T* = (6–80) K) calorimetric experiments. The “AKSAMIT” data acquisition system, connected with a personal computer, performed the control of the measurement process, as well as the determination of the heat capacity values.

The calorimeter reliability was verified by the heat capacity measurements of the standard reference materials (benzoic acid C_6_H_5_COOH, corundum α-Al_2_O_3_, copper Cu) over the range of *T* = 6–350 K [47]. The relative expanded uncertainties for the heat capacity (*U*_r_(*C*_p_) = 0.02 below *T* = 15 K, *U*_r_(*C*_p_) = 0.005 between *T* = 15 and 40 K, and *U*_r_(*C*_p_) = 0.002 in the interval of *T* = 40–350 K) were evaluated on the basis of the calibration results.

#### 2.2.2. Differential Scanning Calorimetry

A differential scanning calorimeter DSC 204 *F1 Phoenix* (NETZSCH) was used to determine the heat capacity of hybrid dendrimer G1{Si}_13_[Ar]_32_ in the temperature range *T* = 350–600 K. The calorimeter design and the experimental procedure were described in detail elsewhere [48]. The DSC 204 *F1 Phoenix* calorimeter was calibrated using the high-purity (>99.99%) reference samples, namely, indium (In), tin (Sn), bismuth (Bi), mercury (Hg), biphenyl ((C_6_H_5_)_2_), and cyclohexane (C_6_H_12_), recommended by the IUPAC Technical Report [49]. The calibration experiments were performed at a heating rate of 5 K·min^−1^ in an argon atmosphere with a gas flow of 25 mL·min^−1^. The heat capacity of the studied hybrid dendrimer was calculated using the ratio approach with synthetic sapphire (α-Al_2_O_3_) as a reference [50]. The dendrimer mass loaded into the DSC crucible was 12.53 mg. The DSC measurements were carried out in the interval of *T* = 350–600 K at a heating rate of 5 K·min^−1^ under an argon purge (25 mL·min^−1^). The NETZSCH *Proteus* software was used to analyze the obtained DSC results. As a result, the standard uncertainty for the temperature *u*(*T*) = 0.2 K and the relative expanded uncertainty for the heat capacity *U*_r_(*C*_p_) = 0.02 were determined in the range of *T* = 350–600 K.

## 3. Results and Discussion

### 3.1. Heat Capacity

**Figure 4:**
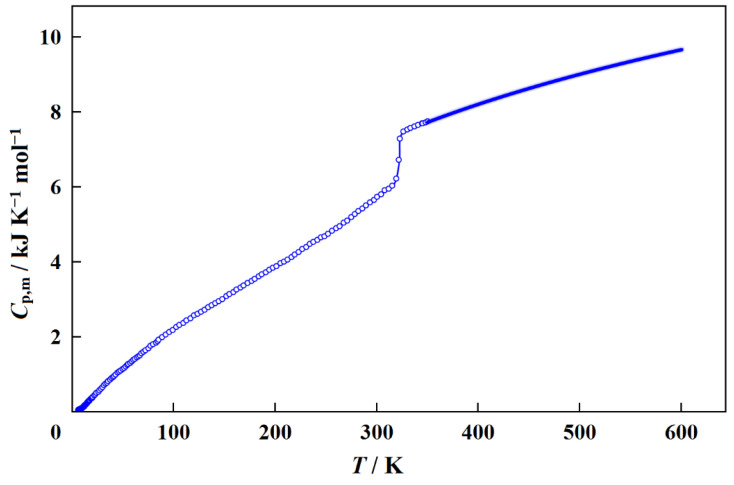
Temperature dependence of the molar heat capacity of hybrid dendrimer G1{Si}_13_[Ar]_32_ (circles **○** (6–350 K) and the solid line—(350–600 K) correspond to measurements in adiabatic calorimeter and DSC, respectively).

The temperature dependence of the molar heat capacity of the hybrid dendrimer with a “carbosilane core/phenylene shell” structure G1{Si}_13_[Ar]_32_ is presented in Figure 4. The *C*_p,m_ values of the studied dendrimer in the experimental temperature range *T* = 6–600 K are listed in Table S1 (Supplementary Materials). The mass loss of hybrid dendrimer G1{Si}_13_[Ar]_32_ was not observed after the calorimetric experiments, and this is in good agreement with the results of TG analysis. The heat capacity of hybrid dendrimer G1{Si}_13_[Ar]_32_ was not more than 25–60% of the total heat capacity of the calorimetric ampoule filled with the substance.

### 3.2. Standard Thermodynamic Characteristics of the Glass Transition and the Glassy State

The studied hybrid dendrimer G1{Si}_13_[Ar]_32_ was devitrified during the heat capacity measurements in the interval of *T* = 300–350 K. The glass transition temperature $T_{g}^{o}$ = 323 ± 1 K was determined using the method of Alford and Dole [51]. The heat capacity increase at the glass transition temperature $\Delta C_{p}^{o}(T_{g}^{o})$ = 1312 ± 16 J·K^−1^·mol^−1^ was evaluated graphically. The configuration entropy $S_{\mathrm{conf}}^{o}$ = 334 ± 5 J·K^−1^·mol^−1^ was calculated using Equation (1) [52].

(1) $$S_{\mathrm{conf}}^{o}=\Delta C_{p}^{o}(T_{g}^{o})\cdot \ln(T_{g}^{o}/T_{K}^{}),$$

where *T*_K_ is the Kauzmann temperature [53], and the ratio $\left(T_{g}^{o}/T_{K}^{}\right)$ = 1.29 ± 0.14 [54]. The assumption $S_{\mathrm{conf}}^{o}$ ≈ *S*°(0) was used to determine the absolute entropy *S*°(*T*) of dendrimer G1{Si}_13_[Ar]_32_.

**Figure 5:**
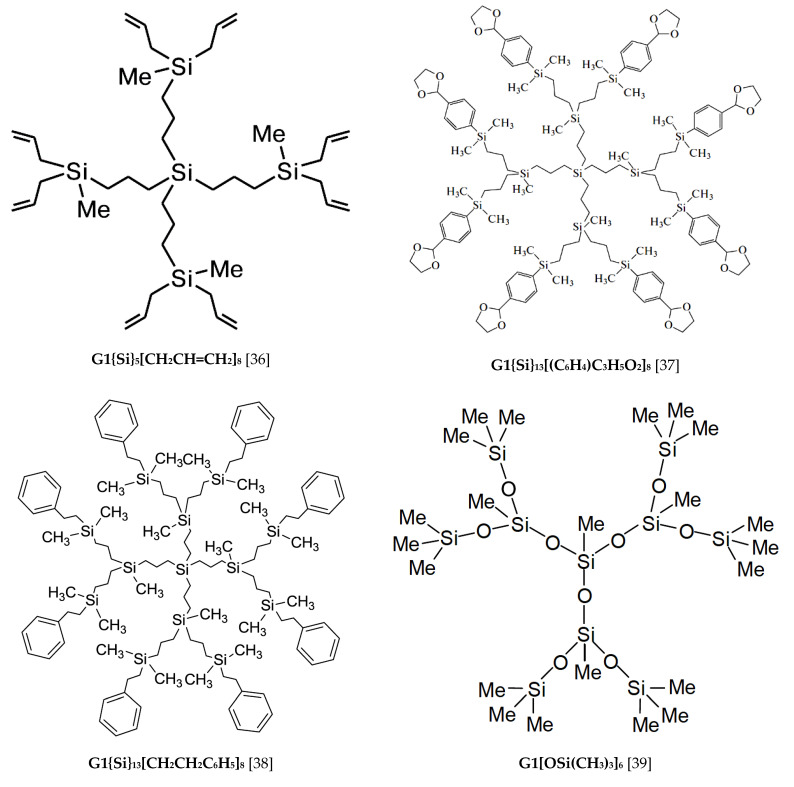

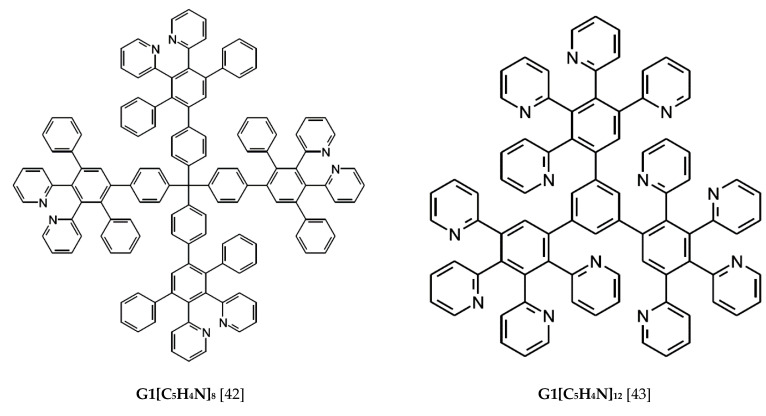
Molecular structures of the calorimetrically studied organosilicon and pyridylphenylene dendrimers of the first generation.

The standard thermodynamic characteristics of the glass transition and glassy state of the studied hybrid dendrimer G1{Si}_13_[Ar]_32_ are presented in Table 2 in comparison to the data for the investigated organosilicon [35,40,41,42] and pyridylphenylene [45,46] dendrimers of the first generation. Molecular structures of the calorimetrically studied G1 dendrimers are shown in Figure 5.

As can be seen from Table 2, the studied hybrid dendrimer G1{Si}_13_[Ar]_32_ was characterized by the highest glass transition temperature in comparison to the entirely carbosilane dendrimer G1{Si}_5_[CH_2_CH=CH_2_]_8_ [36] and siloxane dendrimer G1[OSi(CH_3_)_3_]_6_ [39]. Furthermore, the studied hybrid dendrimer had the highest $S_{\mathrm{conf}}^{o}$ value among the other dendrimers of the first generation. The configuration entropy can characterize the mobility of the individual macromolecule parts. Thus, the $S_{\mathrm{conf}}^{o}$ can take a minimum value or is equal to zero for strictly ordered systems. In our case, the value of $S_{\mathrm{conf}}^{o}$ = 334 J·K^−1^·mol^−1^ for hybrid dendrimer G1{Si}_13_[Ar]_32_ may indicate a disordered structure of the dendritic macromolecule. The intermediate $T_{g}^{o}$ values were revealed in case of carbosilane dendrimers G1{Si}_13_[(C_6_H_4_)C_3_H_5_O_2_]_8_ [37] and G1{Si}_13_[CH_2_CH_2_C_6_H_5_]_8_ [38] containing the cyclic phenyl and heterocyclic dioxolane units on the surface layer. The previously studied pyridylphenylene dendrimer G1[C_5_H_4_N]_12_ [43] had exactly the same $T_{g}^{o}$ value as the investigated hybrid dendrimer G1{Si}_13_[Ar]_32_. Moreover, the absolutely identical behavior of the heat capacity curves can be explained by the similar structure of these macromolecules due to the presence of rigid phenylene fragments. As a result, the glass transition temperature of dendrimers with different molecular skeleton and functional terminal groups largely depends on the structure of their periphery layer.

### 3.3. Standard Thermodynamic Functions

The standard thermodynamic functions of hybrid dendrimer G1{Si}_13_[Ar]_32_ were calculated in the interval of *T* = 6–600 K. The graphical extrapolation of the low-temperature *C*_p,m_ values to absolute zero was performed in accordance with the Debye *T*^3^ law [55].

(2) $$C_{p}^{o}=nD(\Theta {}_{D}/T),$$

where **D** is the Debye function, and *n* and Θ**_D_** are specially selected parameters (*n* = 30, Θ**_D_** = 49.0 K). The experimental *C*_p,m_ values of the investigated dendrimer were described by Equation (2) with the relative expanded uncertainty *U*_r_(*C*_p,m_) = 0.014.

The standard thermodynamic functions (the enthalpy (*H*°(*T*) − *H*°(0)), the entropy (*S*°(*T*) − *S*°(0)), and the Gibbs energy (*G*°(*T*) − *H*°(0))) of dendrimer G1{Si}_13_[Ar]_32_ are listed in Table 3. The calculation procedure was described in detail elsewhere [56,57].

The standard entropy of formation Δ_f_*S*° of dendrimer G1{Si}_13_[Ar]_32_ was calculated at a reference *T* = 298.15 K using the value of (*S*°(*T*) − *S*°(0)) = 5865 J·K^−1^·mol^−1^ (Table 3), the residual entropy *S*°(0) = 334 J·K^−1^·mol^−1^ (Table 2), and the absolute entropies of the elemental substances C_(*gr*)_ (*S*° = 5.74 J·K^−1^·mol^−1^), H_2(*g*)_ (*S*° = 130.68 J·K^−1^·mol^−1^), and Si_(*cr*)_ (*S*° = 18.82 J·K^−1^·mol^−1^) recommended by Chase [58]. The obtained Δ_f_*S*° value,

Δ_f_*S*°(G1{Si}_13_[Ar]_32_, 298.15) = −(17,484 ± 97) J·K^−1^·mol^−1^,

corresponds to the following formation reaction of dendrimer:

304C_(*gr*)_ + 166H_2(*g*)_ + 13Si_(*cr*)_ → C_304_H_332_Si_13(*gl*)_,

where the physical states are given in parentheses (*gr*—graphite, *g*—gas, *cr*—crystal, *gl*—glassy state).

## 4. Conclusions

In this work, we reported the results of a calorimetric study of the first-generation hybrid dendrimer with a “carbosilane core/phenylene shell” structure. The temperature dependence of the heat capacity of the studied compound was determined in the range of *T* = 6–600 K by precise adiabatic calorimetry and DSC. The glass transition of hybrid dendrimer G1{Si}_13_[Ar]_32_ was observed in the experimental temperature interval. The standard thermodynamic characteristics of this transformation were determined and compared with those of the studied earlier organosilicon and pyridylphenylene dendrimers with different functional terminal groups. The standard thermodynamic functions of hybrid dendrimer G1{Si}_13_[Ar]_32_ were calculated over the range *T* = 0–600 K. The standard entropy of formation of the studied compound was evaluated at *T* = 298.15 K. It was confirmed that the structure of the surface layer of dendrimers strongly influences their thermal behavior. The rigid phenylene shell considerably increases the glass transition temperature of hybrid dendrimers and makes them more thermally stable macromolecules than carbosilane and siloxane dendrimers.

## Figures and Tables

**Table 1:** Sample information.

Designation of Dendrimer	Brutto Formula	Source	Purification Method	Final Mass Fraction Purity	Analysis Method
G1{Si}_13_[Ar]_32_	C_304_H_332_Si_13_	Synthesis [44]	Gel permeation chromatography	0.98	Elemental analysis, NMR spectroscopy, MALDI-TOF mass spectrometry

**Table 2:** The standard thermodynamic characteristics of the glass transition and glassy state of the first-generation dendrimers with different molecular skeleton and functional terminal groups at *p*° = 0.1 MPa *^a^*.

Type of Dendrimer	Designation of Dendrimer	Δ*T* (K)	$T_{g}^{o}\pm \;1\;(K)$	$\Delta C_{p}^{o}(T_{g}^{o})\;(J\cdot K^{-1}\cdot {\mathrm{mol}}^{-1})$	$S_{conf}^{o}\approx \;S{}^{\circ}(0)\;\;(J\cdot K^{-1}\cdot {\mathrm{mol}}^{-1})$	Source
Hybrid	G1{Si}_13_[Ar]_32_	300–350	323	1312 ± 16	334 ± 5	This work
Carbosilane	G1{Si}_5_[CH_2_CH=CH_2_]_8_	150–160	154	406 ± 5	103 ± 2	[36]
	G1{Si}_13_[(C_6_H_4_)C_3_H_5_O_2_]_8_	200–260	231	1180 ± 11	301 ± 3	[37]
	G1{Si}_13_[CH_2_CH_2_C_6_H_5_]_8_	176–215	198	960 ± 10	245 ± 3	[38]
Siloxane	G1[OSi(CH_3_)_3_]_6_	137–153	147	245 ± 3	63 ± 1	[39]
Pyridylphenylene	G1[C_5_H_4_N]_12_	290–350	323	225 ± 3	57 ± 1	[43]

*^a^* The standard uncertainty for pressure *u*(*p*) = 10 kPa. The reported expanded uncertainties correspond to the 0.95 level of confidence (coverage factor *k* ≈ 2).

**Table 3:** The standard thermodynamic functions of hybrid dendrimer G1{Si}_13_[Ar]_32_ (*M*(C_304_H_332_Si_13_) = 4350.95 g·mol^−1^) at *p*° = 0.1 MPa *^a^*.

*T* (K)	$C_{p}^{o}(T)\;(\mathrm{kJ}\cdot K^{-1}\cdot {\mathrm{mol}}^{-1})$	(*H*°(*T*) − *H*°(0)) (kJ·mol^−1^)	(*S*°(*T*) − *S*°(0)) (kJ·K^−1^·mol^−1^)	−(*G*°(*T*) − *H*°(0)) (kJ·mol^−1^)
*Amorphous (glassy) state*				
5	0.0196	0.0249	0.00667	0.00838
10	0.0948	0.298	0.04161	0.118
15	0.224	1.08	0.103	0.468
20	0.3689	2.569	0.1879	1.189
30	0.6452	7.638	0.3897	4.053
40	0.9112	15.46	0.6129	9.056
50	1.142	25.73	0.8412	16.33
60	1.359	38.23	1.068	25.88
70	1.572	52.87	1.294	37.69
80	1.798	69.74	1.519	51.75
90	2.005	88.69	1.741	68.04
100	2.196	109.7	1.963	86.56
150	3.034	240.9	3.015	211.4
200	3.862	413.8	4.004	387.1
250	4.692	628.1	4.957	611.1
298.15	5.675	877.0	5.865	871.6
300	5.706	887.6	5.900	882.5
320	6.070	1006	6.282	1004
323	6.100	1022	6.331	1020
*Amorphous (devitrified) state*				
323	7.412	1022	6.331	1020
330	7.534	1076	6.499	1068
350	7.736	1229	6.949	1203
400	8.216	1628	8.014	1577
450	8.638	2050	9.006	2003
500	9.016	2491	9.936	2477
550	9.358	2951	10.81	2996
600	9.671	3427	11.64	3557

*^a^* The standard uncertainty for pressure *u*(*p*) = 10 kPa. The standard uncertainties for temperature *u*(*T*) = 0.01 K in the interval of *T* = 5–350 K and *u*(*T*) = 0.5 K in the range of *T* = 350–600 K. The combined expanded relative uncertainties *U*_c,r_($C_{p}^{o}(T)$) = 0.02, 0.005, 0.002, and 0.02; *U*_c,r_([*H*°(*T*) − *H*°(0)]) = 0.022, 0.007, 0.005, and 0.022; *U*_c,r_([*S*°(*T*) − *S*°(0)]) = 0.023, 0.008, 0.006, and 0.023; *U*_c,r_([*G*°(*T*) − *H*°(0)]) = 0.03, 0.01, 0.009, and 0.03 in the intervals of *T* = 5–15 K, *T* = 15–40 K, *T* = 40–350 K, and *T* = 350–600 K, respectively. The reported expanded uncertainties correspond to the 0.95 level of confidence (coverage factor *k* ≈ 2).

## Data Availability

Data are contained within the article and Supplementary Materials.

## Supplementary Materials

The following are available online at https://www.mdpi.com/article/10.3390/e23121557/s1, Experimental values of the molar heat capacity of hybrid dendrimer G1{Si}_13_[Ar]_32_ (Table S1).
