# Supplementary material for: Thermodynamic Properties of the First-Generation Hybrid Dendrimer with “Carbosilane Core/Phenylene Shell” Structure
Source: Entropy (Basel). 2021 Nov 23;23(12):1557. doi: 10.3390/e23121557 (PMC8700160; doi:10.3390/e23121557)
Supplement: Supplementary file 1 [file entropy-23-01557-s001.zip › entropy-1456102-supplementary.pdf]

Thermodynamic properties of the first generation hybrid dendrimer with "carbosilane core / phenylene shell" structure

Supplementary Material

Table S1. The experimental molar heat capacity of the first generation hybrid dendrimer $G1\{Si\}_{13}[Ar]_{32}$ ($M(C_{304}H_{332}Si_{13})$ = 4350.95 g mol^{-1}) at p° = 0.1 MPa[a]

T / K	$C_{p,m}$ / J K^{-1} mol^{-1}	T / K	$C_{p,m}$ / J K^{-1} mol^{-1}	T / K	$C_{p,m}$ / J K^{-1} mol^{-1}
Series 1 (AC)*		16.79	275.8	65.40	1471
6.36	38.4	17.20	287.6	66.91	1510
6.47	39.9	17.54	298.3	68.80	1544
6.61	42.0	18.06	313.5	70.65	1590
6.78	44.5	18.47	325.7	72.71	1634
7.01	47.7	18.92	342.9	75.18	1692
7.16	49.8	19.46	354.2	77.66	1749
7.37	52.5	19.91	366.4	80.15	1801
7.59	55.6	20.77	389.9	82.66	1852
7.78	58.2	22.03	426.3	84.43	1888
7.97	60.6	23.34	462.4	*Series 2* (AC)	
8.18	63.5	24.69	499.8	83.27	1863
8.43	67.3	26.12	538.7	85.51	1914
8.59	69.8	27.40	573.5	86.70	1941
8.82	73.4	28.81	612.6	88.93	1984
8.98	75.9	30.17	650.2	89.22	1989
9.25	80.3	31.56	689.1	91.75	2036
9.46	84.0	33.01	728.7	92.41	2049
9.64	87.4	34.42	764.8	95.89	2124
9.90	92.5	36.13	810.4	99.36	2184
10.05	95.6	37.45	847.2	102.84	2253
10.38	103	38.95	883.2	106.33	2311
10.74	111	40.46	924.1	109.82	2371
11.15	121	42.04	960.8	111.50	2403
11.61	132	43.55	996.2	114.89	2454
11.77	136	45.16	1035	118.30	2510
11.92	140	46.65	1068	121.71	2567
12.25	148	48.17	1100	123.80	2606
12.63	159	49.74	1137	127.29	2664
13.04	170	51.32	1173	130.80	2722
13.46	181	52.93	1206	134.31	2781
13.87	193	54.50	1238	137.83	2834
14.20	202	56.11	1273	141.38	2896
14.69	215	57.68	1306	144.90	2946

15.10	227	59.29	1345	148.42	3007
15.59	240.8	60.90	1375	151.94	3069
15.94	251.0	62.47	1409	155.46	3127
16.33	262.4	64.13	1445	158.98	3189

Table S1 – *Continued*

T / K	$C_{p,m}$ / J K^{-1} mol^{-1}	T / K	$C_{p,m}$ / J K^{-1} mol^{-1}	T / K	$C_{p,m}$ / J K^{-1} mol^{-1}
162.51	3255	319.40	6213	440.3	8558
166.04	3311	320.36	6357	443.3	8582
169.57	3367	321.66	6796	446.3	8606
173.12	3430	322.97	7284	449.3	8630
176.67	3489	326.56	7469	452.3	8654
180.23	3549	330.23	7535	455.3	8678
183.79	3605	333.92	7562	458.3	8702
187.35	3667	337.60	7601	461.3	8725
190.93	3723	341.30	7648	464.3	8748
194.51	3778	344.99	7689	467.3	8771
198.19	3836	345.59	7696	470.3	8794
201.79	3886	348.09	7718	473.3	8817
205.38	3952	350.58	7743	476.3	8840
208.99	3998	*Series 3* (DSC)[b]		479.3	8863
212.59	4057	350.3	7736	482.3	8885
216.21	4121	353.3	7767	485.3	8907
219.83	4184	356.3	7798	488.3	8929
223.46	4260	359.3	7828	491.3	8951
227.10	4334	362.3	7858	494.3	8973
230.71	4393	365.3	7887	497.3	8995
234.35	4461	368.3	7917	500.3	9016
238.01	4520	371.3	7946	503.3	9038
241.67	4582	374.3	7975	506.3	9059
245.34	4643	377.3	8003	509.3	9081
249.02	4678	380.3	8032	512.3	9102
252.70	4741	383.3	8060	515.3	9123
256.56	4815	386.3	8088	518.3	9143
260.25	4897	389.3	8116	521.3	9164
263.95	4956	392.3	8143	524.3	9185
267.65	5037	395.3	8171	527.3	9205
271.36	5091	398.3	8198	530.3	9226
275.07	5186	401.3	8225	533.3	9246
278.76	5275	404.3	8251	536.3	9266
282.47	5339	407.3	8278	539.3	9286
286.17	5415	410.3	8304	542.3	9306
289.87	5494	413.3	8330	545.3	9326
293.56	5584	416.3	8356	548.3	9345

297.25	5659	419.3	8382	551.3	9365
300.95	5721	422.3	8408	554.3	9385
304.62	5801	425.3	8433	557.3	9404
308.28	5896	428.3	8458	560.3	9423
310.25	5918	431.3	8484	563.3	9442
311.93	5943	434.3	8508	566.3	9461
315.76	6019	437.3	8533	569.3	9480

Table S1 – *Continued*

T / K	$C_{p,m}$ / J K^{-1} mol^{-1}	T / K	$C_{p,m}$ / J K^{-1} mol^{-1}	T / K	$C_{p,m}$ / J K^{-1} mol^{-1}
572.3	9499	413.5	8332	509.5	9082
575.3	9518	416.5	8358	512.5	9103
578.3	9537	419.5	8384	515.5	9124
581.3	9555	422.5	8410	518.5	9145
584.3	9574	425.5	8435	521.5	9166
587.3	9592	428.5	8460	524.5	9186
590.3	9611	431.5	8485	527.5	9207
593.3	9629	434.5	8510	530.5	9226
596.3	9647	437.5	8535	533.5	9247
599.3	9665	440.5	8559	536.5	9267
600.3	9671	443.5	8584	539.5	9287
Series 4 (DSC)		446.5	8608	542.5	9307
353.5	7769	449.5	8632	545.5	9327
356.5	7800	452.5	8656	548.5	9346
359.5	7830	455.5	8680	551.5	9366
362.5	7859	458.5	8703	554.5	9386
365.5	7889	461.5	8727	557.5	9405
368.5	7918	464.5	8750	560.5	9424
371.5	7947	467.5	8773	563.5	9443
374.5	7976	470.5	8796	566.5	9462
377.5	8005	473.5	8819	569.5	9481
380.5	8034	476.5	8841	572.5	9501
383.5	8062	479.5	8864	575.5	9519
386.5	8090	482.5	8886	578.5	9538
389.5	8117	485.5	8909	581.5	9556
392.5	8145	488.5	8931	584.5	9575
395.5	8172	491.5	8953	587.5	9593
398.5	8200	494.5	8975	590.5	9611
401.5	8226	497.5	8996	593.5	9630
404.5	8253	500.5	9018	596.5	9648
407.5	8280	503.5	9039	599.5	9666
410.5	8306	506.5	9061	600.5	9672

* AC – adiabatic calorimetry, DSC – differential scanning calorimetry.

[a] The standard uncertainty for pressure $u(p)$ = 10 kPa. The standard uncertainties for temperature $u(T)$ = 0.01 K in the interval of T = (6.33–350.58) K, $u(T)$ = 0.5 K in the range of T = (350.3–600.3) K. The relative expanded uncertainties for the molar heat capacity $U_r(C_{p,m})$ = 0.02, 0.005, 0.002, and 0.02 in the intervals of T = (6.33–15.10) K, T = (15.52–40.46) K, T = (41.99–350.58) K, and T = (350.3–600.3) K, respectively. The reported expanded uncertainties correspond to the 0.95 level of confidence (coverage factor $k \approx 2$).

[b] The $C_{p,m}$ values in *Series 3 and 4* (DSC) correspond to the devitrified state of dendrimer. Each third $C_{p,m}$ value is given.
